# The Impact of the Ca^2+^-Independent Phospholipase A_2_β (iPLA_2_β) on Immune Cells

**DOI:** 10.3390/biom11040577

**Published:** 2021-04-15

**Authors:** Tayleur D. White, Abdulaziz Almutairi, Ying Gai Tusing, Xiaoyong Lei, Sasanka Ramanadham

**Affiliations:** Department of Cell, Developmental and Integrative Biology, Comprehensive Diabetes Center, University of Alabama at Birmingham, Birmingham, AL 35294, USA; twhite1@uab.edu (T.D.W.); aziiz@uab.edu (A.A.); yingg@uab.edu (Y.G.T.); xlei@uab.edu (X.L.)

**Keywords:** iPLA_2_β, macrophages, T-cells, inflammation, eicosanoids, resolvins

## Abstract

The Ca^2+^-independent phospholipase A_2_β (iPLA_2_β) is a member of the PLA_2_ family that has been proposed to have roles in multiple biological processes including membrane remodeling, cell proliferation, bone formation, male fertility, cell death, and signaling. Such involvement has led to the identification of iPLA_2_β activation in several diseases such as cancer, cardiovascular abnormalities, glaucoma, periodontitis, neurological disorders, diabetes, and other metabolic disorders. More recently, there has been heightened interest in the role that iPLA_2_β plays in promoting inflammation. Recognizing the potential contribution of iPLA_2_β in the development of autoimmune diseases, we review this issue in the context of an iPLA_2_β link with macrophages and T-cells.

## 1. Introduction

Phospholipases A_2_ (PLA_2_s) hydrolyze the *sn*-2 substituent of glycerophospholipids to release a lysophospholipid and a free fatty acid [1]. Among the family of PLA_2_s are the group VI Ca^2+^-independent PLA_2_s (iPLA_2_s), which include iPLA_2_β (VIA), iPLA_2_γ (VIB) iPLA_2_δ (VIC), iPLA_2_ε (VID), iPLA_2_ζ (VIE), and iPLA_2_η (VIF) [2]. The cytosolic iPLA_2_β enzyme, along with the membrane-associated iPLA_2_γ, are the most studied of the group VI PLA_2_s. Because of its emerging link with inflammation, the focus of this review will be on iPLA_2_β.

Encoded by *PLA2G6*, the iPLA_2_β protein (84-88 kDa) has a serine lipase consensus sequence (GTSGT), contains eight N-terminal ankyrin repeats, an ATP binding cassette (GGGVKG), a caspase-3 cleavage site (DVDT), and two calmodulin (CAM) binding domains [2,3]. Crystal structure studies suggest that iPLA_2_β forms a dimer through the interaction of the catalytic domains and that CAM binds to the dimer to cause a closed state, denying the access of substrates to the active site [4]. Relief from this inhibitory state is achieved through activation of calmodulin kinase IIβ, which forms a signaling complex with iPLA_2_β [5]. Lipidomics-based LC/MS approaches have revealed that among glycerophospholipids, iPLA_2_β exhibited the greatest activity towards 1-palmitoyl-2-arachidonoyl-sn-glycero-3-phosphoethanolamine (PAPE) and, among *sn*-2 substituents, a greater selectivity for linoleate or myristate [6,7]. The currently available selective inhibitors of iPLA_2_β include the irreversible *S*-BEL (*S*-bromoenol lactone) and the reversible 1,1,1-trifluoro-6-(naphthalen-2-yl)hexan-2-one (FKGK18) [8,9]. iPLA_2_β is widely expressed and has many proposed roles, including signal transduction, and is recognized to contribute to neurodegenerative disorders, cancers, myocardial complications, and metabolic dysfunction (reviewed extensively elsewhere [2,3]).

While localized in the cytosol under basal conditions [10], iPLA_2_β mobilizes to subcellular organelles (e.g., the endoplasmic reticulum (ER), mitochondria, nucleus) upon stimulation. Its activation leads to the hydrolysis of the *sn*-2 fatty acid substituent from membrane glycerophospholipids [11,12,13,14,15,16] to yield a free fatty acid (e.g., arachidonic acid, eicosapentaenoic acid (EPA), docosahexaenoic acid (DHA)) and a 2-lysophospholipid [17]. Subsequent metabolism of arachidonic acid by cyclooxygenases (COX), lipoxygenase (LOX), and cytochrome P450 (CYP) pathways leads to the generation of bioactive oxidized eicosanoids, several of which are proinflammatory [18] and recognized contributors to inflammatory diseases [19,20,21,22,23,24,25,26,27]. Some of the most potent inflammatory eicosanoids [21] are prostaglandin E_2_ (PGE_2_), leukotrienes (LTs), hydroxyeicosatetraenoic acids (HETEs), and dihydroxyeicosatetraenoic acids (DHETEs), and they contribute to inflammation and autoimmune diseases [22]. It is not unexpected that iPLA_2_β activation can play critical roles in the initiation and progression of inflammatory pathways, which if not curbed can lead to the evolution of a variety of disorders. In contrast, other unsaturated fatty acids such as EPA and DHA can be metabolized to generate pro-resolving lipids, designated as specialized resolving mediators (SPMs) [28,29,30]. Among the proinflammatory lipids generated, PGs and LTs are the first to be produced [28]. When there is injury to the tissue, the generated PGs cause pain, swelling, and edema. To resolve the inflammation and clear antigenic debris, SPMs are generated in attempt to restore homeostasis, a process referred to as “lipid mediator class switching” [31,32].

Immune cells express iPLA_2_β [33,34,35,36] and iPLA_2_β-derived lipids (iDLs) contribute to cell proliferation [37], cell cycle progression [38], cell division [39], monocyte migration [40], and superoxide generation [41]. Inhibition of iPLA_2_β reduces reactive oxygen species (ROS) generation [42] and is reported to be effective against autoimmune- [43] and inflammation-based [44,45,46,47] diseases.

Although extensive literature exists linking a number of secretory PLA_2_s (sPLA_2_s) or cytosolic PLA_2_α (cPLA_2_α) with inflammatory responses, only a few studies have described a link between iPLA_2_β and macrophages, and even fewer have considered a link between iPLA_2_β and T-cells or B-cells. To provide context, in this paper, the basic functionality of these cells is discussed first, followed by a review of the specific impact of iPLA_2_β in these immune cells, in the context of different pathophysiologies.

## 2. Macrophages

Macrophages are recognized to play significant roles in the development of autoimmune diseases, including rheumatoid arthritis (RA), multiple sclerosis (MS), and type-1 diabetes (T1D) [48]. As fundamental components of the innate immune system, they contribute to tissue homeostasis, dead cell and antigen phagocytosis, and crucial induction of adaptive immunity [49]. They do so by recognizing pathogen-associated molecular patterns (PAMPs) of foreign microorganism or damage-associated molecular patterns (DAMPs) from damaged host cells through pattern recognition receptors (PRR). PPRs are found in all innate immune cells including macrophages. Following their recognition, macrophages internalize the PAMPs or DAMPs via phagocytosis and break them down for antigen presentation through major histocompatibility complex II (MHC-II) [50], which is another fundamental role of macrophages [51]. T lymphocytes recognize self-peptides that are presented on the MHC Class-II molecules and expressed mainly by antigen presenting cells (APC) like dendritic cells and macrophages [52]. All tissues have resident macrophages, which emerge from three different developmental stages—the yolk sac, fetal liver, and bone marrow [53,54]. Although fetal-liver and bone marrow-derived monocytes are the main source of macrophages after birth, yolk sac-derived macrophages give rise to microglia and a small fraction of tissue-derived macrophages [55].

Macrophages are essential in both inflammation and healing processes. However, the functionality of macrophages is subject to macrophage polarization, defined as the “M1-M2 paradigm” [56]. Tissue-resident macrophages are mainly of the M2 (anti-inflammatory) phenotype [57] and affect tissue homeostasis and the resolution of inflammation, which also involves debris clearance [58]. Polarization of macrophages to the M2 phenotype is induced by Th2 cytokines, including interleukins 4, 13, and 10 (IL-4, IL-13, and IL-10). This leads to upregulation of arginase-1 (Arg1), chemokine (C-C motif) ligands 17 and 24 (CCL17 and CCL24), and secretion of IL-10 and transforming growth factor beta (TGFβ) [59]. In contrast, the M1 macrophages express a proinflammatory phenotype and are induced by Th1 cytokines that include interferon gamma (IFNγ) and TNFα. This promotes upregulation of Arg2, iNOS, cyclooxygenase-2 (COX-2), and other proinflammatory factors, leading to increased production of nitric oxide (NO), TNFα, IL-1α, IL-1β, IL-6, and IL-12 [60]. Whereas M2-like macrophages represent the majority of tissue-resident macrophages [57,61], M1-like macrophages are found significantly in inflamed tissues [60].

Emerging studies highlight the importance of lipids in macrophage functionality [62,63]. Although macrophage signaling has long been studied in the context of cytokine and chemokine production, recent studies suggest a profound impact of eicosanoids and SPMs on macrophage functions [31,64]. Eicosanoids have been linked to modifying macrophage inflammatory response in multiple diseases [23,65,66]. In humans, PGE_2_ induces LOX-class switching from leukotriene B4 (LTB_4_) to lipoxins, which represents a stop signal for polymorphonuclear (PMN) recruitment and initiation of a resolution phase that promotes an anti-inflammatory macrophage phenotype and function (i.e., phagocytosis) [30]. Facilitating the resolution process are SPMs, which counter-regulate the early initiators (PGs and LTs) of acute inflammation, leading to inhibition of proinflammatory cytokines and upregulation of anti-inflammatory cytokines (e.g., IL-10) [29].

## 3. T-Cells and B-Cells

Lymphocyte development in mammals occurs in the central lymphoid organs such as the bone marrow (B-cell development) and the thymus (T-cell development) [67]. From these organs, T and B lymphocytes continue to migrate to other peripheral lymphoid tissues. Differences in both lymphocyte populations occurs in mature stages, where T-cell development slows down in the thymus, and they divide outside of the central lymphoid organs to maintain the number of mature T-cells [67].

The development of a cluster of either differentiation 4 or differentiation 8 (CD4^+^ or CD8^+^) T-cells depends on the thymocyte maturation process. This involves hematopoietic stem cells in the bone marrow migrating to the thymus, hence the “T” in T-cells, to mature into functional T-cells [68]. The earliest thymocytes are double-negative—they do not express either CD4 or CD8. In the double negative (DN) stage, these cells are negative for T-cell receptor (TCR) expression, are not able to bind to MHC, and do not carry out effector functions. The DN stage occurs in the cortex and has four subsets that are important for the full development of T-cells, designated as DN1–4. DN3 is a critical step in T-cell development and encompasses: (1) thymocytes becoming restricted to a T-lineage and generation of mature αβ and γδ T-cells, (2) T-cell receptor β-chain (TCRβ) *V(D)J* recombination, (3) expression of functional pre-TCR complex, and (4) thymocytes becoming early pre-T-cells that are committed to the αβ T-cell lineage. The DN4 cells start to express low levels of CD4 and CD8 and present themselves as double-positive (DP) [68]. During the DP stages, CD4 and CD8 expression levels are upregulated and the cells migrate to the medulla, where a negative selection process confers single positivity for CD4 or CD8.

Naïve CD4 T-cells are destined for four distinct fates that are determined by different cytokine patterns after interaction with antigens. These cytokine signals upregulate distinct transcription factors to generate specific cell populations, which include the T helper cells 1, 2, and 17 (Th1, Th2, Th17), and regulatory T-cells (Tregs) [69].

B lymphocytes also go through different stages, including (1) activation, (2) selection, and (3) maturation. Like T lymphocytes, B-cells are derived from hematopoietic stem cells (HSCs) that go through stages in the bone marrow. These stages include the pro-B-cell (progenitor B-cell), the pre-B-cell (precursor B-cell), the immature naïve B-cell, and lastly the mature naïve B-cell stage [70]. After maturation, the naïve B-cells migrate to a secondary lymphoid tissue (e.g., lymph nodes), where they can be activated by antigens to generate memory B cells and plasma antibody-secreting cells.

T and B lymphocytes are major cellular components for autoimmunity. B-cells are responsible for antigen presentation, antibody production, and cytokine production. However, T-cells take part in both “helper” and cytotoxic activity. T-helper cells, also known as CD4^+^ T-cells, are a type of T-cells that play an essential role in adaptive immune responses. Cytotoxic T-cells, CD8^+^ T-cells, are identified as T-cells that kill cancer cells or infected cells.

Both CD4 and CD8 T lymphocyte types contribute to the adaptive immunity component of the immune system. There are two distinct types of immunity based on B- and T-cell function: humoral and cell-mediated. Humoral immunity is based on the antibodies produced by plasma cells, which are derived from mature naïve B-cells. This allows for an individual exposed to a disease to be administered antibodies already generated by another individual previously exposed to the same disease. Cell-mediated immunity, however, involves T-cells, specifically the cytotoxic (CD8) T-cells. Similar to humoral immunity, T-helper and cytotoxic cells from an individual who was exposed to and survived a disease are administered to an individual who has been freshly exposed to the same disease. The helper T-cells are able to activate other immune cells and cytotoxic T-cells, initiating the removal process of those pathogens [71]. Hence, both humoral and cell-mediated immunity result in the binding of the antibodies to the antigen and affect their elimination.

## 4. Immune Cells and Lipids

Proinflammatory lipids promote CD4 Th1/Th17 differentiation [72], inhibit Tr1 differentiation [72], induce NF-κB [73], inhibit Treg cell differentiation [73], modulate local activation of T-cells [74], induce NO [75], participate in oxidative stress pathways [76], increase the expression of cytokine genes and CCL2, also known as monocyte chemoattractant protein-1 (MCP-1) [77,78], amplify ER stress [79,80,81], and reduce inflammation-resolving processes [82,83,84,85]. Furthermore, reduced cellular debris clearance [86] and apoptotic clearance defects in macrophages and dendritic cells of non-obese diabetic (NOD) mice, an autoimmune spontaneous T1D-prone model, have been attributed to PGE_2_ [87], which is significantly higher in infiltrated versus insulitis-free NOD islets [74]. Other lipids, in particular lysophosphatidic acid (LPA), are powerful chemoattractants [40,42,88,89]. LOX products induce migration of leukocytes, p38 mitogen-activated protein kinase pathway, ROS-generating NADPH oxidase (NOX), proinflammatory cytokine production, and β-cell apoptosis [90,91,92,93,94,95]. 12-LOX-deficiency protects mice from streptozotocin (STZ)-induced T1D [96] and reduces insulitis and T1D incidence in the NOD [97]. 12-LOX is detectable in islet cells in recent-onset T1D but absent in non-diabetic subjects [98].

Several publications over the years have introduced the concept that lipid mediators and fatty acids influence immune responses [99,100]. One of the most relevant T-cell discoveries has been the characterization of signaling platforms enriched in cholesterol and glycosphingolipids, which are named lipid rafts. These rafts are important for downstream signaling pathways for enzymes, scaffold proteins, and adaptors [101].

## 5. Deleterious Impact of iPLA_2_**β** Activation

### 5.1. Cellular Compass

**Figure 1:**
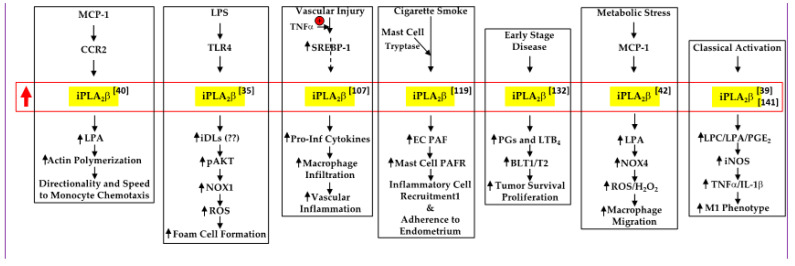
Proposed mechanisms by which iPLA_2_β activation contributes to monocyte chemotaxis, foam cell formation, vascular and bladder inflammation, tumor cell survival, and macrophage polarization. LPA, lysophosphatidic acid; iDLs, iPLA_2_β-derived lipids; ROS, reactive oxygen species; EC, endothelial cell (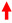, increased expression/activity of iPLA_2_β; 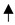, indicates increase in).

Immune responses are propagated through the recruitment of peripheral blood leukocytes to inflamed areas. The chemokine CCL2 plays a critical role in this process by signaling through receptor CC chemokine receptor 2 (CCR2) [102]. The Cathcart group, examining the role of CCL2 in promoting directed migration of monocytes, identified distinct roles for iPLA_2_β and cPLA_2_α [40,103]. Upon CCL2 stimulation, cPLA_2_α translocated from the cytosol to the endoplasmic reticulum (ER) and regulated monocyte migration via arachidonic acid and its metabolites. In contrast, iPLA_2_β was recruited to the membrane-enriched pseudopod. Here, iPLA_2_β was proposed to catalyze hydrolysis of the *sn*-2 fatty acid from PLD-derived phosphatidic acid (PA) [104]. This phospholipid is among the negatively charged lipid substrates preferred by iPLA_2_β [7,105], and leads to the accumulation of LPA. Utilizing the iPLA_2_β-selective inhibitor (*S*-BEL) [106] and antisense oligodeoxyribucleotide (ODN), the authors demonstrated that iPLA_2_β-generated LPA regulates actin polymerization to facilitate directionality to the migrating monocytes. They go on to suggest that iPLA_2_β may manifest a cellular compass role or be an integral component of the cellular compass. Consistently, lipids derived from iPLA_2_β activation, but not other PLA_2_s, have been demonstrated to promote monocyte chemotaxis [42,88,107,108] (Figure 1).

### 5.2. Foam Cell Formation

Macrophages contribute to atherosclerosis development, and it requires their conversion to lipid-laden foam cells via a toll-like receptor (TLR)-mediated process [109]. Lipopolysaccharide (LPS) plays a critical role in this process through the generation of ROS [110,111]. Lee et al. [35] demonstrated that LPS binding to TLR4 induces NOX1 expression and, as a consequence, ROS production. Both *S*-BEL and small interfering RNA (siRNA) directed against iPLA_2_β, but not *R*-BEL or siRNA directed against membrane-associated iPLA_2_gamma (iPLA_2_γ), were found to decrease NOX1 expression, and consequently, ROS production. This was associated with mitigation of foam cell formation. They further reported that iPLA_2_β effects are signaled through the Akt pathway, and although the specific lipid signal involved was not identified, they hypothesized that iDLs promote Akt phosphorylation. However, this remains to be elucidated.

### 5.3. Vascular Injury

Neointima formation leads to several vascular-related pathologies and macrophages are critical contributors to vascular inflammation [112]. Liu et al. [107] using *S*-BEL and siRNA directed against iPLA_2_β, iPLA_2_β-deficient mice, and mice that selectively overexpress iPLA_2_β in smooth muscle cells, demonstrated that iPLA_2_β participates in ligation-induced neointima formation. They further demonstrated that this was associated with increased production of proinflammatory cytokines and vascular infiltration by macrophages. By comparing the effects of inhibiting the arachidonic acid metabolism pathways COX2 with indomethacin, 5-LOX with NDGA, 12-LOX with baicalein, CYP with 17-octadecynoic acid, and 12/15-LOX with luteolin, they deduced that the products of 12-LOX and 15-LOX are complicit in the formation of neointima. In view of reports suggesting that sterol regulatory element-binding protein 1 (SREBP-1) expression is induced in the injured vascular wall [95] and SREBP-1 induces iPLA_2_β [113,114], they speculated that SREBP-1-mediated induction of iPLA_2_β occurs in their model of ligation-induced neointima formation. Interestingly, they found that the overexpression of iPLA_2_β in the smooth muscle cells does not promote neointima formation and that it is only amplified upon injury, leading to increased TNFα production. As basally iPLA_2_β is inactivated through an interaction with calmodulin [5,115,116], they postulate that TNFα causes disassociation of the complex to unmask iPLA_2_β activity. Analogously, under basal conditions, β-cells overexpressing iPLA_2_β do not exhibit a difference in death rate; however, upon stimulation with ER stressors or pro-inflammatory cytokines, they exhibit exacerbated apoptosis [117,118].

### 5.4. Cigarette Smoke

An interesting link between iPLA_2_β and platelet activating factor (PAF) was recognized by McHowat’s group in their study of smoke-induced bladder inflammation [119]. This appears to involve a series of events initiated by the tethering of inflammatory cells to the apical endothelial cell surface, rolling of the inflammatory cells across the endothelium and tight adherence, and subsequent PAF-mediated transmigration between neighboring endothelial cells [120,121]. One route of PAF production, predominant during inflammation, is via remodeling initiated by the hydrolysis of the *sn*-2 substituent from phosphatidylcholine by PLA_2_ activation to generate alkyl-lysophosphatidyl choline (LPC). Subsequently, an acetyl group is added at the *sn*-2 position to generate PAF. McHowat’s group observed that upon exposure to cigarette smoke, human and rodent bladder endothelial cells (ECs) exhibited higher PAF accumulation, decreased activity of platelet-activating factor acetylhydrolase (PAF-AH), which degrades PAF, and increased inflammatory cell adherence. These outcomes were reversed by chemical inhibition or genetic knockout of iPLA_2_β, and by PAF receptor (PAFR) antagonism. Cellular adhesion is facilitated by adhesion molecules such as platelets/endothelial cells (P/E)-selectin and intercellular adhesion molecule 1 (ICAM-1) and these are produced by mast cells [120], which are key contributors to chronic inflammation. Mast cells express PAFR and also release tryptase, which can induce iPLA_2_β activation in bladder epithelial cells [122]. The authors proposed that mast-cell-induced PAF generation by endothelial cells via iPLA_2_β activation can prolong the inflammatory state, thus leading to chronic inflammation. Of interest, Ueno et al. [123] reported that cPLA_2_α, but not iPLA_2_β, contributes to arachidonic acid production by bone marrow-derived mast cells, facilitating their maturation. Curiously, although mast cells have been reported not to be involved in the development of T1D, which is a consequence of autoimmune destruction of β-cells, in the NOD rodent model [124], they have been suggested to be potentially important in promoting β-cell dysfunction and death in human T1D [125].

### 5.5. Early-Stage Disease

A common blood cancer is chronic lymphocytic leukemia (CLL), which is characterized by immune-incompetent B-CLL lymphocytes [126,127]. These cells express COX2 and have increased production of PGE_2_, PGF_2_, and LTB_4_. The latter signals through the BLT1 receptor to activate CD40-dependent chronic B lymphocytic leukemia cells to prolong B-CLL survival [128,129]. Greater expression of PLA_2_ enzymes has been noted in tumor cells, relative to healthy cells, supporting their role in tumor cell proliferation and metastasis [38,130,131]. The work of Guriec et al. suggests that both cPLA_2_α and iPLA_2_β are expressed in the tumor cells, with cPLA_2_α expression increasing higher in patients with advanced-stage disease and with iPLA_2_β expression being higher in the early disease stages [132]. The disease progression was observed to be associated with dysregulation of arachidonic acid metabolizing pathways (COX, 5-LOX) and of LTA4 hydrolase (LTA4H), which generates LTB_4_. The progressive increases in PGs and LTB4, the latter of which activates its receptors BLT1 and BLT2, are proposed to support tumor cell proliferation and survival.

### 5.6. Metabolic Stress

It is well recognized that type 2 diabetes (T2D) is associated with increased coronary artery disease and atherosclerosis [133] and that these involve recruitment of macrophages to inflamed vascular sites [134]. Additionally, cellular oxidative stress appears to be a compounding factor. ROS are generated as byproducts of oxygenases or primary products of NOX. Among the NOX variants [135], although both Nox4 and Nox2 are expressed in macrophages [136], Nox4 promotes monocyte chemotaxis and macrophage recruitment during diabetic metabolic stress (DMS) [137]. Tan et al. [42] reported that under conditions mimicking DMS (i.e., high glucose and LDL), overexpression of iPLA_2_β amplified macrophage NOX4 expression, ROS production, and CCL2-induced migration. Conversely, these outcomes were mitigated by *S*-BEL and siRNA directed against iPLA_2_β. The same interventions had no effect on NOX2. Interestingly, NOX2 is localized primarily in the plasma membrane, whereas NOX4 is localized in the mitochondria, ER and nuclear membranes [138,139], which are also the subcellular organelles that iPLA_2_β mobilizes to upon the induction of stress [11,12,14,15,140]. Tan et al. further suggested that iPLA_2_β-derived LPA manifested these effects, as LPA receptor antagonism prevented NOX4 induction and LPA addition was able to rescue outcomes inhibited by *S*-BEL.

### 5.7. Macrophage Polarization

In view of the reported impact of iPLA_2_β on macrophage function, we sought to identify the role of iDLs in this process [39,141]. We found that iPLA_2_β activation induces macrophage polarization, favoring the M1 inflammatory phenotype. Utilizing selective inhibitors, we identified that both COX and LOX products contribute to iPLA_2_β-modulated macrophage polarization. Such an outcome was mitigated in macrophages from iPLA_2_β-deficient mice upon classical activation (LPS+IFNγ). This was reflected by reductions in M1 markers (*Arg2* and *Nos2*) and increases in several M2 markers, including *Arg1* and *Ccl2*. Interestingly, *Ptgs2* and *Alox12*, which encode COX2 and 12-LOX, also decreased, suggesting feedback regulation by products of these enzymes. Among the iDLs identified as inducers of the M1 phenotype were 6-keto PGF_1_α, PGE_2_, and LTB_4_. The inhibition of secretory PLA_2_s (sPLA_2_s) GIIA, GV, and GX or cPLA_2_α did not alter the classically activated macrophage production of eicosanoids, suggesting a select role for iDLs on macrophage polarization. However, other groups have reported a role for sPLA_2_ or cPLA_2_α in eicosanoid production from macrophages [142,143,144,145,146,147]. Those studies were performed with macrophage-like cells (RAW264.7 and P388D1) or peritoneal macrophages from rats and mice, with only LPS (10-1000 ng/mL) or zymosan stimulation for 6–48 h, and the involvement of various PLA_2_s was discerned with chemical inhibitors, some of which effected multiple PLA_2_s. In contrast, our studies were performed with primary mouse peritoneal macrophages, which have been demonstrated to behave differently from the macrophage-like cells [36], from wild-type and genetically- modified iPLA_2_β-deficient mice, and importantly, treated with LPS+IFNγ, conditions that induce a M1 macrophage phenotype. Interestingly, while iPLA_2_β-deficiency skewed macrophage polarization towards an M2 anti-inflammatory phenotype, inhibition of downstream oxygenases did not necessarily promote an M2 phenotype. We posit that a reduction in macrophage-iPLA_2_β activity, as a consequence of an attenuated inflammatory landscape, promotes a shift to a recovery/repair milieu.

### 5.8. T1D Development

Immune cells play a significant role in promoting β-cell death, which leads to T1D development [148,149]. In T1D-prone individuals, macrophages are among the first immune cells that migrate to pancreatic islets to initiate inflammatory responses and secrete proinflammatory cytokines and ROS, which lead to β-cell death [150,151]. Two different activation states of macrophages have been described—M1 proinflammatory macrophages [152], which are classically activated (e.g., by IFNγ, LPS, TNFα); and M2 anti-inflammatory macrophages, which are alternatively-activated (e.g., by IL-4 or IL-10) [153]. Although M1 macrophages are recognized to be causative factors in T1D development [154], M2 macrophages protect against T1D development [155].

Macrophages are the only resident myeloid cells found in pancreatic islets under normal conditions in all mice strains [156]. However, the cellular composition of pancreata from diabetic individuals and NOD mice revealed a considerable presence of macrophage infiltration into pancreatic islets, with a predominant M1-like phenotype [33,156,157]. Macrophages found in close contact with β-cells present diabetogenic antigens, predominantly insulin peptides [158,159,160]. The proinflammatory M1 macrophages produce ROS, which, along with proinflammatory cytokines such as IL-1β, IFNγ, and TNFα, cause β-cell destruction [49,161]. It is well-recognized that macrophages work in concert with other immune cells to promote β-cell destruction in T1D [149,162,163]. Furthermore, proinflammatory eicosanoids are linked to macrophage phagocytosis, adhesion, apoptosis, and amplifying macrophage-derived eicosanoid release [24,164,165,166]. In the context of T1D, 12-LOX-null NOD has a lowered T1D incidence, which is associated with reduced macrophage production of proinflammatory cytokines [97].

It is also well-established that in human T1D patients and NOD mice, both CD4 and CD8 T-cells are the major components of the islet infiltrate [162]. Different stages of disease onset show varying compositions of CD4 and CD8 T-cells, depending on the timeframe of progression. First, autoreactive T-cells are activated by β-cell antigens presented by antigen-presenting cells (APCs). The activated T-helper cells are required to activate CD8 T-cells. Second, the activated CD4 T-cells infiltrate the pancreas and are thought to contribute to β-cell destruction via the activation of macrophages [154].

CD4 T-cells recognize MHC class II peptides presented by APCs in order to carry out β-cell destruction. CD4 T-cell-mediated β-cell destruction can be caused by the production of proinflammatory cytokines such as IFNγ, which are toxic to the β-cell, and indirectly, by activating local innate cells such as macrophages and dendritic cells (DCs) to enhance infiltration [154]. CD4 T-cells are also thought to interact with CD8 T-cells by facilitating their activation [154]. CD8 T-cells can directly kill β-cells by interacting with MHC class I molecules and by means of perforin and granzyme secretion [149]. It has been suggested that the MHC class I/CD8 T-cell interaction is required for T1D in the early stages of development [162] and antigen presentation to CD4 T-cells within pancreatic islets is essential for β-cell destruction [154]. Other reports have suggested that the MHC class I/CD8^+^ T-cell interaction is required for T1D in the early stages of development [167] and that antigen presentation to CD4 T-cells within pancreatic islets are essential for β-cell destruction [154]. Taken together, the literature establishes that immune cells work in concert to promote β-cell destruction in T1D [21].

**Figure 2:**
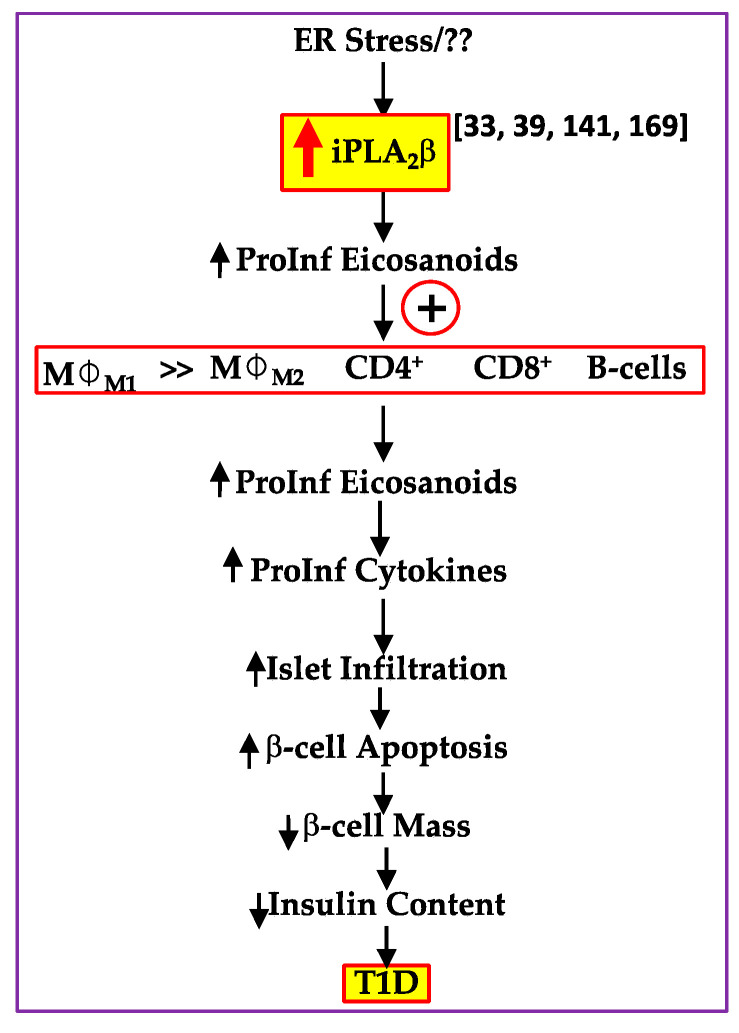
Proposed contribution of iDLs generated by immune cells to β-cell destruction and type-1 diabetes (T1D) development. (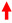, increased expression/activity of iPLA_2_β; 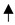, indicates increase in; and 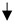, indicates decrease in).

Evidence linking iDLs with T1D development (Figure 2). Several reports have provided evidence of an association between deleterious outcomes in experimental and clinical diabetes and iPLA_2_β activation [41,168].

In view of these observations, we explored the possibility that inhibition of iPLA_2_β can ameliorate T1D [33,169]. We found that administration of FKGK18 to female NOD mice promoted several positive outcomes. There was a significant reduction in insulitis, as reflected by reductions in islet abundances of CD4^+^ T-cells and B-cells. Glucose homeostasis was also improved, as reflected by β-cell preservation and higher circulating insulin. Consequentially, a significant reduction in T1D was achieved. Inhibition of iPLA_2_β resulted in decreased production of TNFα from CD4^+^ T-cells and antibodies from B-cells, suggesting that iDLs modulate immune cell responses. This was supported by the recapitulation of the mitigated TNFα production by select iPLA_2_β inhibitors, with COX and 12-LOX inhibition. TNFα acts as a powerful chemoattractant [89] and is produced by CD4 T-cells within inflamed islets during T1D development [170]. TNFα overexpression exacerbates insulitis, whereas the opposite occurs in TNFβ-receptor-null mice [171]. To date, ours are the first and only reports of the modulation of T- and B-cell functions by iDLs. Similar findings in a genetically modified NOD model with reduced iPLA_2_β expression [169] further support this possibility.

These findings prompted us to further examine iDL production by macrophages. Not surprisingly, a dramatically more profound proinflammatory landscape was evident in macrophages from the NOD, relative to the spontaneous diabetes-resistant C57BL/6J, mouse [169]. Lipidomic assessments in the NOD model identified select iDLs (PGE_2_, PGD_2_, hydroxyeicosatetraenoic acids 5 and 15 (5-HETE and 15-HETE), LTC_4_) that correlated with T1D development [169]. Importantly, a similar lipid signature was revealed in the plasma of human subjects at high risk of developing T1D [169]. We therefore posit that select iDLs contribute to T1D onset and that these could be targeted for therapeutics and, in conjunction with autoantibodies, serve as early biomarkers of pre-T1D.

## 6. Protective Consequences of iPLA_2_**β** Activation

### 6.1. Cancer Development

**Figure 3:**
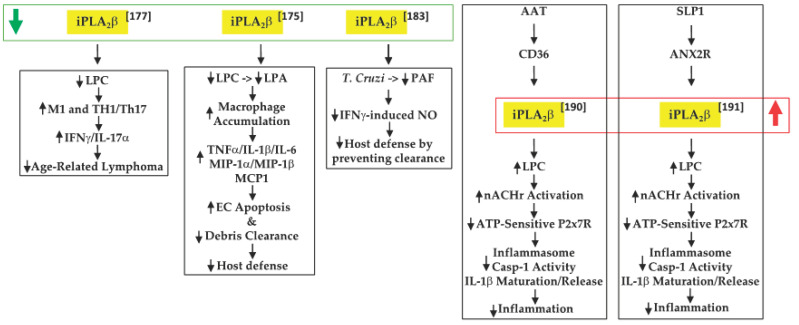
Proposed mechanisms by which iPLA_2_β inactivation or activation can have protective outcomes in tumor development and promoting host defenses. (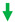, decreased expression/activity of iPLA_2_β; 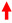, increased expression/activity of iPLA_2_β; 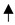, indicates increase in; 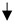, indicates decrease in).

Inflammation is a key contributor to cancer development [172] and cytokines released by macrophages and T-cells are integral to this process [173]. In view of their earlier observations that iPLA_2_β-null mice are more susceptible to various inflammatory-based disorders [174,175,176], Inhoffen et al. [177] assessed the ability of immune cells from iPLA_2_β-null mice to produce cytokines following exposure to CD95/FasL, a trigger of proinflammatory cytokine production [178]. They found that iPLA_2_β-deficiency increased apoptosis in the liver, spleen, and mesenteric lymph nodes (MLN). Although Kupffer cells (i.e., satellite macrophages in the liver) generated a lower production of proinflammatory cytokines TNFα and IL-6, splenocytes became primed to release proinflammatory Th1-/Th17-related cytokines (IFNγ/IL-17α). These findings led to the suggestion that iPLA_2_β-deficiency can reduce age-related MLN lymphoma development. Mechanistically, they attributed this to the decreased availability of “find-me” and “eat-me” signals derived via iPLA_2_β activation. These include LPC, a find-me signal [88], which promotes the clearance of apoptotic debris, the accumulation of which triggers and amplifies subsequent immune responses [179,180] (Figure 3).

### 6.2. Inflammatory Bowel Disease (IBD)

An inflammatory disorder such as IBD is a consequence of the dysfunction of the intestinal epithelial barrier and the mucosal immune system [181]. Jiao et al. [175] further explored the role of “find-me” signals derived via iPLA_2_β activation in the context of dextran sodium sulfate-induced IBD. They found that iPLA_2_β-deficiency promotes the accumulation of infiltrating macrophages and dendritic cells in the colon lamina. These were associated with increased production of inflammatory cytokines (TNFα, IL-1β, and IL-6), macrophage inflammatory proteins (MIP-1α and MIP-1β), and CCL2, leading to intestinal epithelial cell apoptosis and mucus barrier damage. With a concurrent decrease in LPC levels, they speculated that iPLA_2_β-deficiency mitigated the availability of a “find-me” signal, which limited the clearance of apoptotic debris and the amplification of immune responses. Further, they predicted that the iPLA_2_β-deficiency also reduced LPA levels, impacting the cellular compass, and preventing the optimal phagocytic function of macrophages. Subsequently, Murase et al. [182], comparing the involvement of various PLA_2_s, suggested that iPLA_2_β-deficiency did not worsen the clinical scoring associated with IBD, relative to controls. As they did not reconcile the two studies, it is likely that differences between the source of the iPLA_2_β^−/−^ mice and DSS concentrations used may be partly responsible. Furthermore, while a 7-day DSS regimen was employed by both groups, Jiao’s group maintained the mice for an additional 3 days without DSS, prior to analyses. It may also be noted that Murase’s study did demonstrate similar lower clinical scores in the wild-type (WT) and iPLA_2_β-deficient groups between days 1 and 6, relative to mice with deficiencies in cPLA_2_α or in a variety of sPLA_2_s; however, at day 7, the scores in the iPLA_2_β-deficient group appeared to be as high as in the other PLA_2_-deficient groups. As such, the role of iPLA_2_β in this model remains to be clarified.

### 6.3. Chagas Disease

A further link between iPLA_2_β and PAF was reported by McHowat’s group [183], in the context of Chagas disease. This disease is caused through infection by the protozoan parasite [184] *Trypanosoma cruzi (T. cruizi)* and can lead to various cardiac abnormalities [185]. The sequela of infection begins with induction of an inflammatory response and upregulation of endothelial adhesion molecules [186], followed by attempts at resolution through the generation of proinflammatory cytokines and the induction of signaling pathways to promote the chemotaxis of immune cells to mitigate the invasion. McHowat’s group reported that infection of iPLA_2_β-deficient mice resulted in lowered PAF and NO production by cardiac endothelial cells, but that neither the expression of adhesion molecules nor the development of myocardial inflammation was affected. However, significant increases in parasite pseudocysts were noted in the myocardium of iPLA_2_β-deficient mice. The authors suggested that this was due to an impairment in parasite clearance as a consequence of decreased iPLA_2_β-mediated LPA production and, as a result, Nox4 expression and NO production. Thus, they surmised that the absence of iPLA_2_β mitigates parasite clearance due to the reduced recruitment of inflammatory cells to the infected myocardial areas.

### 6.4. Negative Modulation of Inflammation by AAT and SLP1

In sequential reports, Grau’s group constructed events that participated in regulating IL-1β activation and release. IL-1β is critical to host defense against infections, but the generation of excessive active IL-1β can lead to deleterious inflammatory consequences [187]. The release of IL-1β occurs via two signals. The first is an external stimulus that induces the synthesis of pro-IL-1β. The second signal has been suggested to be ATP, which when released from damaged cells activates the purinergic receptor P2 × 7R, promoting the loss of K^+^ current and triggering the assembly of NLRP3 (NLR family pyrin domain containing 3)-containing inflammasome [188]. This leads to caspase-1 activation, cleavage of pro-IL-1β, and release of IL-1β from the immune cell. Present in inflammatory cells, alpha-1 antitrypsin (AAT) is a strong inducer of anti-inflammatory processes and its upregulation during systemic inflammation has been associated with the decreased production of proinflammatory cytokines, including IL-1β [189]. In examining the potential role of AAT in ATP-dependent regulation of IL-1β in their first study [190], Siebers et al. found that AAT signaling through the CD36 receptor activates iPLA_2_β, which leads to the release of a low molecular weight factor (LMWF). The LMWF is released from the cell and binds to nicotinic acetylcholine receptor (nAchR), leading to inhibition of P2X7R function, prevention of inflammasome assembly, and the processing of pro-IL-1β to active IL-1β, and its release from the cell. These outcomes were significantly mitigated with selective inhibition or knockdown of iPLA_2_β and were also not evident in PBMCs from iPLA_2_β-deficient mice. In the second study [191], Zakrzewicz et al. demonstrated that the secretory leukocyte protease inhibitor (SLP1) also interferes with ATP-dependent regulation of IL-1β via iPLA_2_β activation. SLP1 is also present in inflammatory cells [192] and its actions promote anti-inflammatory outcomes. Their results suggested that the SLP1 signals released through annexin 2, a membrane binding protein for SLP1 [193], activate iPLA_2_β, leading to the production of the LMWF and subsequent inhibition of IL-1β maturation and release. Although both pathways were found to mitigate inflammation, unfortunately neither study explored the identity of the LMWF. Interestingly, AAT administration to recent-onset T1D subjects improved β-cell function, which was correlated with reduced IL-1β production from monocytes and myeloid dendritic cells [194].

## 7. Summary

iPLA_2_β is a member of the family of PLA_2_s that hydrolyzes the *sn*-2 substituent from membrane glycerophospholipids. Thus, activation of iPLA_2_β can lead to the production of a variety of bioactive lipid mediators. As eicosanoids generated subsequently to iPLA_2_β-mediated hydrolysis of *sn*-2 arachidonic acid can exhibit profound proinflammatory effects and SPMs are produced from *sn*-2 substituents EPA and DHA, the impact of iPLA_2_β on the inflammatory sequelae is profound. In recent years, the impact of iPLA_2_β at the immune cell level is being recognized and those studies, as well as those from our laboratory, suggest that iPLA_2_β activation modulates the maturation, polarization, activation, and functionality of macrophages, T-cells, and B-cells. The continuation of studies addressing these actions of iPLA_2_β is important and warranted in order to gain a better understanding of the events that lead to the onset, maintenance, and amplification of inflammation. Identifying the relevant selective iDLs with effects on these processes could lead to the development of new strategies to treat autoimmune- and other inflammatory-based diseases.

## Data Availability

Not Applicable.
